# Learning Challenges and Motivations of Medical Students in Japan's Regional Quota System: A Qualitative Study From Okinawa Prefecture

**DOI:** 10.7759/cureus.95688

**Published:** 2025-10-29

**Authors:** Miyu Yara, Richi Kakazu, Yuiko Hiyajo, Hirotake Machida, Kiyoshi Kinjo, Ryuichi Ohta

**Affiliations:** 1 Family Medicine, Faculty of Medicine, University of the Ryukyus, Okinawa, JPN; 2 Medical Education, Faculty of Medicine, University of the Ryukyus, Okinawa, JPN; 3 Community Care, Unnan City Hospital, Unnan, JPN

**Keywords:** education, health workforce, medical, medical students, motivation, qualitative research, rural health services, undergraduate

## Abstract

Introduction

Japan's Regional Quota System (Chiiki-Waku) aims to mitigate physician shortages in medically underserved areas by enrolling medical students who are committed to providing regional service. However, students in this system face distinct academic and emotional challenges, particularly in geographically and culturally unique regions like Okinawa Prefecture. Despite the system's importance, there is limited research exploring these students' educational experiences.

Methods

This qualitative study examined the motivations, challenges, and learning experiences of 12 Chiiki-Waku medical students at the University of the Ryukyus. Semi-structured interviews were conducted and analyzed using a thematic analysis approach. Researchers engaged in iterative coding and theme development, ensuring credibility through reflexive discussion and peer debriefing.

Results

Three overarching themes and 11 related concepts were identified from the interviews. Theme 1 was "Motivation stemming from a sense of obligation and inferiority", including pre-admission academic anxiety, efforts to overcome feelings of inferiority, fear of repeating a year, and commitment reinforced by loan obligations. Theme 2 was "Uncertainty and narrow perspectives resulting from changes in the Regional Quota System", covering inadequate information, feelings of confinement, emotional strain, and conflict between duty and personal aspirations. Theme 3 was "Motivation and learning fostered by interpersonal connections", highlighting inspiration from diverse activities, supportive relationships, and shared peer-learning systems. Together, these themes illustrate how quota-based students navigate tension between institutional constraints and personal growth through obligation, uncertainty, and connection.

Conclusion

Chiiki-Waku students in Okinawa demonstrate strong commitment and adaptability despite systemic and psychosocial challenges. Tailored support systems, transparent policy communication, and structured mentorship should be strengthened to enhance educational outcomes and retention in underserved areas. These findings inform educational reforms that can better support regional medical workforce development across Japan.

## Introduction

The Chiiki-Waku system, also known as Japan's Regional Quota System, is a framework established to secure healthcare professionals in medically underserved areas [1]. This system is implemented in various forms across Japan, with medical students admitted under these quotas obliged to contribute to regional healthcare [2,3]. However, while the system aims to address physician shortages, students enrolled through the Regional Quota System often encounter unique learning environments and challenges [4]. Despite this, there remains a limited understanding of the specific difficulties these students experience and how these challenges influence their academic and professional development.

Examining students' learning experiences in the Regional Quota System is crucial for understanding the diversity of medical education and the obstacles that arise due to system-related differences [5,6]. Chiiki-Waku students in Okinawa Prefecture face distinctive circumstances, as the region consists of a chain of widely dispersed inhabited islands, leading to geographical isolation and limited access to centralized educational resources. In addition, cultural characteristics, such as local dialects and community-specific expectations, may affect students' adaptation to clinical training and professional practice. Understanding how these geographic and cultural factors shape their learning and growth may help in designing more effective educational strategies and support systems tailored to their needs.

However, the current state and learning challenges faced by students enrolled in the Regional Quota System may not be fully understood, especially in terms of how variations in the system impact their educational experiences. In Okinawa Prefecture, the quota program requires a nine-year service obligation after graduation, with restrictions and recommendations regarding specialties such as general medicine, internal medicine, and pediatrics. These institutional characteristics may shape students' motivations and learning in distinctive ways. The findings may inform improvements in academic policies and support mechanisms, ultimately contributing to the sustainability of regional healthcare by ensuring that these students receive adequate preparation for their future roles [1,2,7].

Previous research has provided limited insights into the academic and professional challenges faced by students in the Regional Quota System, particularly in Okinawa Prefecture [8]. Studies addressing the specific difficulties Chiiki-Waku students encounter in this region are scarce, leaving a significant gap in the literature [9,10]. Thus, this study aims to clarify the challenges that Chiiki-Waku students in Okinawa Prefecture face during their medical education and to bridge the knowledge gap by examining the experiences of these students and identifying areas for potential intervention.

## Materials and methods

Setting

This qualitative study, employing thematic analysis, explored the motivations and factors influencing the decision to adopt the Regional Quota System (Chiiki-Waku) at the Faculty of Medicine, University of the Ryukyus, the only national medical faculty in Okinawa Prefecture. Each year, the faculty admits about 110 students, of whom approximately 17 are enrolled under the Chiiki‑Waku program (14 regional quota and three remote/island quota students). These students receive loan-based scholarships from the prefectural government, which cover tuition and living expenses. They are obligated to serve as physicians for 9-14 years after graduation, including four years of service in remote or northern areas of the prefecture. These institutional characteristics define the study's setting and provide essential context for understanding the unique experiences and motivations of Chiiki-Waku students.

Study population

The participants comprised 12 regional quota (Chiiki-Waku) medical students enrolled in the first to fourth years at the Faculty of Medicine, University of the Ryukyus. Participants were recruited through purposive sampling to ensure variation in academic year, gender, and hometown (main island versus remote island). Recruitment was facilitated via faculty announcements and student peer networks. Students who self-identified as being enrolled under the regional quota program and were willing to share their experiences were eligible.

Semi-structured interviews

To gain a deeper understanding of the challenges and motivations for learning among students in the Regional Quota System and the system's impact on their career choices and perspectives, the interview guide was structured around the central topic: "The challenges and overcoming as students in the Regional Quota System."

The semi-structured interview included open-ended questions that encouraged reflection on both personal and institutional experiences. The concrete questions explored participants' perceptions of what they had learned during medical school and how the Regional Quota System influenced their academic life and plans. Examples of key interview questions included the following: "What motivated you to apply for the Regional Quota (Chiiki-Waku) program?", "How has being a Chiiki-Waku student influenced your learning experiences or daily life at the university?", "Can you describe any challenges you have faced as a Chiiki-Waku student, and how you have tried to overcome them?", "How do the scholarship or service obligations affect your motivation or career planning?", and "In what ways have your relationships with peers, teachers, or community members shaped your learning or professional aspirations?".

Questions also addressed specific factors such as the influence of academic performance, financial incentives, family or community encouragement, and personal values, including the desire to serve remote or underserved areas. The interview guide remained flexible, allowing the interviewer to probe further based on participants' responses.

Analysis

All interviews were audio-recorded, transcribed verbatim, and analyzed using thematic analysis as described by Braun and Clarke [11]. Transcripts were organized and coded using NVivo 14 (QSR International, Melbourne, Australia) to facilitate systematic data management, code retrieval, and comparison across cases. The study followed six systematic phases: (1) familiarization with the data, (2) initial coding, (3) generation of preliminary themes, (4) review and refinement of themes, (5) definition and naming of themes, and (6) interpretation in context. To ensure analytical rigor and reflexivity, the research team, which included six authors with complementary roles, collaborated throughout the process. Four members (RK, HM, MY, and YH) were medical students at the University of the Ryukyus, three of whom were Chiiki-Waku students. They conducted and transcribed the interviews, bringing insider perspectives to the data. RO, a family physician and public health researcher experienced in qualitative analysis, and KK, a senior medical educator overseeing the Chiiki-Waku program, provided external and supervisory perspectives to balance potential bias. During familiarization, all team members independently read the transcripts multiple times to immerse themselves in the data. In coding, the four student researchers independently identified meaningful data segments and developed initial codes inductively. The coding results were then discussed with RO and KK to establish a shared coding framework and ensure consistency across coders. In theme generation, the team collaboratively grouped codes into broader categories that represented recurring patterns or contrasts. These preliminary themes were iteratively reviewed against the transcripts in the refinement phase to confirm alignment with participants' original meanings. Overlapping or ambiguous themes were revised or merged through group discussion. In defining and naming themes, the team agreed on concise, descriptive labels and explanatory narratives. During interpretation, all authors reflected on the themes in relation to the social and institutional context of the Chiiki-Waku program. Consensus was achieved through repeated discussions until thematic saturation was reached. This multi-perspective, iterative approach enhanced the credibility and depth of the analysis, producing themes that authentically captured the complexity of Chiiki-Waku students' motivations and experiences. To visually summarize the analytical process and relationships among the derived themes and concepts, we developed a conceptual figure based on the final stage of thematic interpretation. This figure illustrates how the three overarching themes interact to explain Chiiki-Waku students' motivations, challenges, and coping mechanisms.

Reflexivity

This study was conducted collaboratively, emphasizing the interaction between researchers and participants. The research team comprised individuals from diverse backgrounds and perspectives in rural community care and medical education. Four members of the team (RK, HM, MY, and YH) were medical students at the University of the Ryukyus. Among them, RK, MY, and YH were enrolled under the Regional Quota System, while HM was not. All four were actively involved in a student medical education group focusing on the regional quota, and they participated in conducting the semi-structured interviews. Additionally, RO, a family physician and public health specialist with a master's degree in public health and family medicine, brings extensive research experience in rural community healthcare. KK, a professor at the University of the Ryukyus, oversaw medical education related to the regional quota program. To minimize potential bias, data analysis was conducted through team discussions, where individual interpretations were compared and critically examined. Divergent perspectives were considered, and alternative explanations were explored during the interpretation process to ensure that the findings reflected multiple viewpoints and remained as objective as possible.

Ethical considerations

The Clinical Ethics Committee of Unnan City Hospital approved this study (approval number: 20230039). Participants were provided with detailed explanations about the study's objectives, methods, and privacy protections, and written consent was obtained. Additionally, the collected data were anonymized and guaranteed not to be used for purposes other than research.

## Results

Thematic analysis identified three overarching themes and 11 concepts encapsulating students' learning experiences, emotional struggles, and coping mechanisms in the Regional Quota System (Chiiki-Waku) at the University of the Ryukyus. These results highlight how personal, institutional, and interpersonal dynamics influence their academic journey and professional identity formation (Table 1).

**Table 1:** Themes and concepts regarding students' learning experiences, emotional struggles, and coping mechanisms in the Regional Quota System

Themes	Concept
Motivation stemming from a sense of obligation and inferiority	Pre-admission academic anxiety
	Efforts arising from feelings of inferiority
	Fear of repeating a year as motivation
	Loan obligations enhancing commitment
Uncertainty and narrow perspectives resulting from changes in the Regional Quota System	Inadequate information and fluidity of policies
	Feelings of confinement and narrowed horizons
	Life constraints and negative emotions
	Conflict between duty and personal aspirations
Motivation and learning fostered by interpersonal connections	Inspiration from diverse activities
	Supportive relationships
	Shared learning systems among students

**Figure 1:**
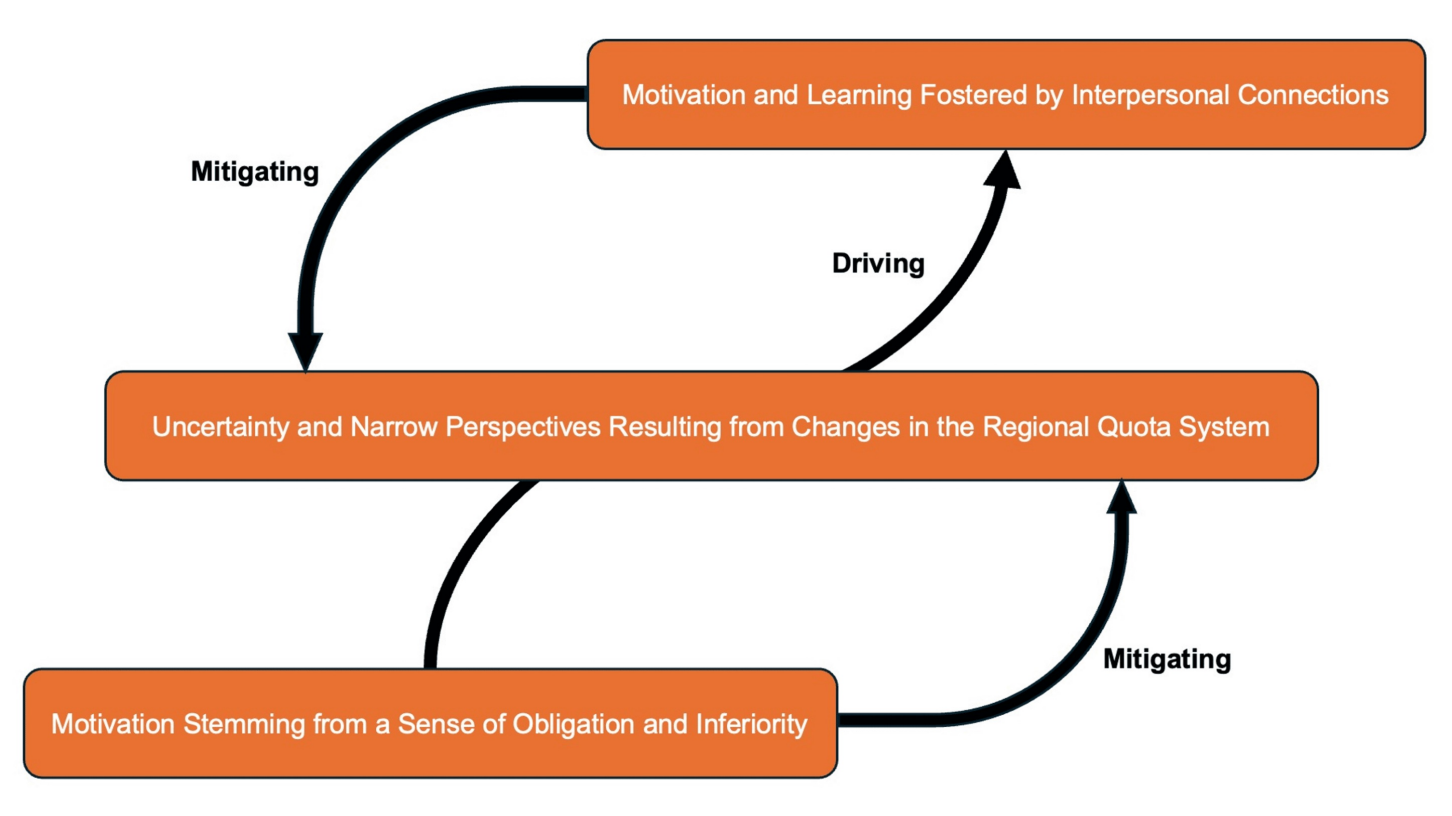
The conceptual figure regarding students' learning experiences, emotional struggles, and coping mechanisms in the Regional Quota System Image Credit: Ryuichi Ohta

The conceptual figure illustrating how the three overarching themes interact to explain Chiiki-Waku students' motivations, challenges, and coping mechanisms is depicted in Figure 1.

Theme 1: motivation stemming from a sense of obligation and inferiority

Pre-admission Academic Anxiety

Many students entered medical school burdened with self-doubt, largely due to the societal perception that the Regional Quota System accepted individuals with lower academic scores. This stigma contributed to a diminished self-perception in academics, even before formal education began. One student reflected, "People probably think I only got in because I didn't have enough points. I even thought my university teachers might believe that" (Participant 6). Such internalized beliefs cultivated a persistent sense of inferiority. However, rather than leading to disengagement, this anxiety often triggered a heightened sense of vigilance and a compensatory drive to validate their legitimacy through academic achievement.

Efforts Arising From Feelings of Inferiority

The perceived academic disparity between regional quota students and their non-quota peers gave rise to ongoing feelings of inferiority, particularly during the early stages of medical education. Many students questioned their intellectual capabilities and worried they were academically unfit for the rigorous environment. One participant shared, "My test scores were low. Honestly, I was really anxious about whether I could handle medical school" (Participant 10). Despite this, students often reframed these insecurities into a personal challenge, using them as fuel to study harder, participate more actively in class, and outperform expectations, demonstrating resilience and a strong desire for self-validation.

Fear of Repeating a Year as Motivation

The prospect of academic failure and repeating a year instilled deep anxiety in many regional quota students. For them, failure was not only a personal setback but also a source of shame and disappointment for those who supported their education, their family members, their mentors, and the local community. As one participant reflected, "Failing a year would cause trouble for my family and mentors. I couldn't let that happen" (Participant 3). This fear of letting others down created a sense of high-stakes responsibility. Moreover, students were aware that failing a year would result in the suspension of their prefectural scholarship, adding a significant financial burden to themselves and their families. This combination of social and financial consequences ultimately served as a compelling external motivator, prompting students to stay on track and work persistently to avoid failure.

Loan Obligations Enhancing Commitment

The scholarship structure tied to the Regional Quota System, which includes a contractual obligation to serve in rural areas, played a pivotal role in shaping students' academic behavior and mindset. Rather than viewing it solely as financial support, many students internalized the commitment as a moral contract with their community. One student remarked, "Because I've accepted this scholarship, I have to stay committed and make sure I succeed" (Participant 6). This sense of duty often translated into sustained motivation and academic discipline. During periods of doubt or fatigue, the obligation served as a compass, reinforcing their educational and professional resolve.

Theme 2: uncertainty and narrow perspectives resulting from changes in the Regional Quota System

Inadequate Information and Fluidity of Policies

Many students described entering the Regional Quota System with limited or unclear information about its structure, expectations, and long-term obligations. Despite efforts to gather resources and seek clarification, the lack of formal guidance and shifting policies created a persistent sense of uncertainty. One participant explained, "I gathered all the documents I could, even searched online, but I never felt I had a full understanding of the quota system" (Participant 1). This ambiguity affected students' ability to make informed decisions about coursework, clinical rotations, and career trajectories, often compounding stress and contributing to a sense of institutional disorientation.

Feelings of Confinement and Narrowed Horizons

Many students described a growing sense of restriction stemming from the quota system's rigid service obligations. The requirement to work in specific rural regions, such as Okinawa, created the impression that their future professional path was already predetermined, leaving little room for exploration or self-direction. As one student reflected, "It felt like my whole future, what hospital I'd work at, when, and where, was already decided, and that was really suffocating" (Participant 1). This perceived lack of autonomy led to emotional strain and a diminished sense of professional freedom, limiting aspirations toward broader specializations or more diverse clinical environments.

Life Constraints and Negative Emotions

The structured nature of the Regional Quota System, while rooted in a mission to address rural healthcare shortages, often imposed psychological burdens on students. Many expressed emotional distress stemming from the perceived loss of personal freedom, especially when career goals or lifestyle preferences clashed with system mandates. One student admitted, "There were times I thought about quitting the quota program, even if it meant repaying the scholarship" (Participant 1). This internal conflict between fulfilling a social responsibility and maintaining personal autonomy led to chronic stress and, in some cases, feelings of burnout and disengagement from medical studies.

Conflict Between Duty and Personal Aspirations

A recurring theme among participants was the struggle to reconcile the obligations of the Regional Quota System with their evolving personal and professional goals. While students often valued the system's mission, they also yearned for opportunities beyond the geographic and specialty constraints it imposed. As one participant candidly noted, "Sometimes I wonder if I should just pay back the loan and leave the quota program so I can explore other options" (Participant 1). This tension created emotional fatigue and uncertainty about the future, with some students contemplating academic leave or career changes to regain a sense of autonomy and direction.

Theme 3: motivation and learning fostered by interpersonal connections

Inspiration From Diverse Activities

Engagement in extracurricular activities such as community outreach, volunteering, and student-led health initiatives played a crucial role in reawakening students' intrinsic motivation for medicine. These experiences often stood in contrast to the rigidity of academic life, offering meaningful encounters that reaffirmed their desire to serve others. One participant shared, "By participating in volunteer and community activities, I remembered why I wanted to become a doctor" (Participant 10). These moments of real-world connection helped restore a sense of purpose, bridging the gap between theoretical learning and human-centered care and reinforcing students' commitment to socially responsive medical practice.

Supportive Relationships

Supportive interpersonal relationships emerged as a vital buffer against the academic and emotional pressures experienced by quota students. Whether through peers navigating similar challenges, seniors offering guidance, or family members providing encouragement, these networks formed a foundation of psychological safety. One student reflected, "Talking with seniors eased my anxiety and motivated me to push forward" (Participant 6). These interactions often served as a reminder that students were not alone in their struggles, fostering resilience, perspective, and motivation. Moreover, such connections helped reinforce a positive professional identity, offering role models and affirming students' belonging within the medical community.

Shared Learning Systems Among Students

Peer-led learning networks were central to supporting academic success and emotional well-being among regional quota students. These informal systems, often formed across year levels, enabled the exchange of study materials, clinical tips, and personal experiences, creating a collaborative learning culture. One participant shared, "We share materials, advice, and stories with each other. It helps me feel I'm not alone" (Participant 5). These grassroots ecosystems alleviated academic pressure and fostered a sense of solidarity and mutual support. They helped normalize shared struggles, enhance confidence, and cultivate a collective resilience crucial for sustained progress.

## Discussion

This qualitative study explored the lived experiences, motivations, and challenges of medical students enrolled in the Regional Quota System in Okinawa Prefecture, Japan. Through a thematic analysis of in-depth interviews, we identified three overarching themes, with 11 underlying concepts, that reveal the multifaceted learning environment shaped by academic pressure, systemic uncertainty, and interpersonal support. These findings provide valuable insights for educators, policymakers, and institutional leaders seeking to enhance the educational experiences and long-term retention of future rural physicians.

A prominent finding was the psychological burden associated with academic self-perception. Students commonly entered the program with pre-admission academic anxiety, primarily fueled by the societal perception that the Regional Quota System accepts individuals with lower academic performance. This stigma, echoed in earlier research on non-traditional admission pathways [12,13], undermined students' confidence and shaped their academic identity in their formative years. However, unlike prior studies that highlighted the risk of disengagement [14], our findings suggest that quota students often convert these feelings of inferiority into motivation for academic excellence. A fear of repeating the previous year and a deception, linked to their scholarships and community expectations, sense of obligation reinforced this internal drive. These motivations align with the literature on extrinsic motivators enhancing academic persistence [15]; however, our data highlight the unique emotional costs associated with such pressure.

Another key theme was the structural and informational instability of the Regional Quota System. Students reported confusion and anxiety due to inconsistent policy communication and vague contractual expectations. The lack of transparent and accessible information created barriers to informed academic and career planning. This echoes existing research on rural recruitment programs, where unclear service obligations and shifting return policies have been linked to reduced retention and increased dissatisfaction [16,17]. In our study, students also expressed emotional distress due to the perceived confinement to Okinawa, resulting in feelings of a narrowed horizon and regret. While the mission of the Regional Quota System, to secure healthcare providers in underserved areas, is well-intentioned and necessary, our findings suggest that a more flexible and transparent implementation is critical to supporting student well-being and fostering long-term commitment.

A significant contribution of this study is its nuanced understanding of the conflict between public duty and personal aspiration. Several students struggled with the tension between their desire to honor their contractual obligations and the wish to pursue diverse or specialized medical careers. This internal conflict occasionally led to thoughts of quitting the program or taking a leave of academic absence. Similar dilemmas have been documented in rural medical placement programs in Australia, Canada, and the United States [18-20], where geographic limitations and restricted career pathways pose challenges to retention despite the provision of financial incentives. Our study highlights the need for structured career counseling, increased flexibility in service locations, and opportunities for postgraduate specialization within the system to address these concerns.

In contrast to these challenges, students also demonstrated considerable resilience and growth through interpersonal connections and community engagement. Peer-led study groups, relationships with senior students, and participation in extracurricular activities played vital roles in maintaining motivation and reinforcing professional identity [21,22]. These grassroots learning networks helped students normalize their struggles, share coping strategies, and cultivate collective academic resilience. The importance of supportive peer environments in medical education is well-established [23-25], and our findings underscore their amplified value in systems that impose additional academic and emotional burdens on students.

Moreover, engagement in community and volunteer activities reignited students' intrinsic motivation, helping them reconnect with their initial desire to contribute to underserved populations [26]. These experiences helped shift students' perspectives from institutional obligation to personal meaning, an essential transition for long-term retention and job satisfaction in rural medicine [27,28]. Future efforts to strengthen quota-based education should embed opportunities for early clinical exposure, mentorship in rural healthcare, and reflection practices that support this identity formation process [29,30].

This study has several limitations. First, it was conducted at a single institution in Okinawa Prefecture, which may limit the transferability of findings to other regional quota programs in Japan or similar systems globally. Cultural and geographic factors unique to Okinawa, such as the dispersion of inhabited islands across a wide area, limited access to centralized healthcare and educational resources, and strong community expectations rooted in local traditions and dialects, may shape students' experiences differently than in mainland contexts. Second, the sample size, though adequate for qualitative analysis, included only 12 participants, which may not capture the full spectrum of experiences among quota students. Third, participants may have underreported negative experiences due to social desirability bias, particularly when discussing institutional policies or support systems. Lastly, while the research team engaged in reflexive discussion to minimize bias, the involvement of researchers with personal ties to the quota system may have influenced interpretation.

## Conclusions

This study provides critical insight into the educational experiences of medical students within Japan's Regional Quota System (Chiiki-Waku). While these students demonstrate resilience and a strong sense of social responsibility, they also face challenges related to societal stigma, academic pressure, policy ambiguity, and geographic constraints. Improving outcomes requires a multifaceted approach that addresses both educational and structural factors. First, establishing formal mentorship programs, linking Chiiki-Waku students with senior peers and rural physicians, can provide ongoing guidance, emotional support, and realistic perspectives on rural practice, thereby enhancing motivation and retention. Second, enhancing information transparency about scholarship conditions, service obligations, and career pathways through orientation sessions and online resources can reduce anxiety and foster trust between students and institutions. Third, developing flexible policy frameworks that allow the partial fulfillment of service obligations across diverse clinical settings (e.g., community hospitals, island clinics, or academic centers) can accommodate students' professional aspirations while sustaining rural health coverage. By integrating these strategies, medical schools and local governments can create a more supportive and adaptive learning environment. Such initiatives not only strengthen students' confidence and professional identity but also improve the long-term sustainability of Japan's rural healthcare workforce.
